# Human Endogenous Retrovirus Expression Is Inversely Associated with Chronic Immune Activation in HIV-1 Infection

**DOI:** 10.1371/journal.pone.0041021

**Published:** 2012-08-07

**Authors:** Christopher E. Ormsby, Devi SenGupta, Ravi Tandon, Steven G. Deeks, Jeffrey N. Martin, R. Brad Jones, Mario A. Ostrowski, Keith E. Garrison, Joel A. Vázquez-Pérez, Gustavo Reyes-Terán, Douglas F. Nixon

**Affiliations:** 1 Center for Research in Infectious Diseases, National Institute of Respiratory Diseases, Mexico City, Federal District, Mexico; 2 Division of Experimental Medicine, Department of Medicine, University of California San Francisco, San Francisco, California, United States of America; 3 Positive Health Program, San Francisco General Hospital, San Francisco, California, United States of America; 4 Department of Epidemiology and Biostatistics, University of California San Francisco, San Francisco, California, United States of America; 5 Department of Immunology, University of Toronto, Toronto, Ontario, Canada; Imperial College London, United Kingdom

## Abstract

Human endogenous retroviruses (HERV) are remnants of ancestral retroviral infections integrated into the germ line, and constitute approximately 8% of the genome. Several autoimmune disorders, malignancies, and infectious diseases such as HIV-1 are associated with higher HERV expression. The degree to which HERV expression *in vivo* results in persistent inflammation is not known. We studied the association of immune activation and HERV-K expression in 20 subjects with chronic, untreated progressive HIV-1 infection and 10 HIV-1 negative controls. The mean HERV-K *gag* and *env* RNA expression level in the HIV-1 infected cohort was higher than in the control group (p = 0.0003), and was negatively correlated with the frequency of activated CD38+HLA-DR+CD4+ T cells (Rho = −0.61; p = 0.01) and activated CD38+HLA-DR+CD8+ T cells (Rho  = −0.51; p = 0.03). Although HIV-infected persons had higher levels of HERV-K RNA expression (as expected), the level of RNA expression was negatively associated with level of T cell activation. The mechanism for this unexpected association remains to be defined.

## Introduction

After infection with human immunodeficiency virus type 1 (HIV-1), CD4^+^ T lymphocytes decline in number at a variable rate unless treatment is started with antiretroviral therapy (ART) [1]. One of the strongest predictors of disease progression is T cell activation. For reasons that remain to be elucidated, the frequency of circulating cells that express CD38 and HLA-DR (both of which are thought be markers of cell activation) predict rate of CD4+ T cell decline and disease progression in both untreated and treated disease; importantly, this effect appears to be independent of viral load [2]–[5]. Patients with advanced HIV-1 disease as measured by low CD4^+^ T cell blood count have the highest levels of T cell activation, concomitant with rapid proliferation and apoptosis [6], [7], enhanced CD4^+^ T cell susceptibility to infection [7]. Chronic cellular immune activation is accompanied by expression of apoptosis markers such as CD95 and PD-1, and by proinflammatory cytokines [8]–[13]. T lymphocytes expressing CD38 show an abnormal cytokine expression [14], [15] suggesting that HIV-1 perturbs cell function as well as cell number. HIV-1-associated inflammation invariably declines during effective antiretroviral therapy, but often remains higher than that observed in uninfected controls [16]. This residual activation has been associated with cardiovascular disease, malignancy, renal disease and accelerated aging [17].

The mechanisms by which HIV-1 infection leads to immune activation have not been completely defined, although several possible pathways have been postulated. Recent attention has focused on the translocation of microbial products from the intestine to the systemic circulation due to a disrupted mucosal barrier [18], [19]. Another potential contributor to T cell activation is reactivation of CD8+ responses specific for latent infections such as cytomegalovirus (CMV) and Epstein-Barr virus (EBV) [20]–[22]. Consistent with this hypothesis is the observation that most activated CD8^+^ T cells are antigen-specific [23].

In this work we examined the possibility that human endogenous retroviruses (HERVs) could also be contributing to T cell immune activation.

HERVs are proviruses of ancestral infections of germ cells that have been incorporated into the genome. About 8% of the genome is comprised of sequences thought to derive originally from these retroviral infections [24], and about 1.1% of the genome is comprised of recognizable open reading frames (ORFs) of retroviral origin [25]. There are several families of HERVs, usually named with one amino acid letter by the host's tRNA assumed to be required for priming reverse transcription at identification time, and encompassing similarities with all retroviral genera except lentiviruses (including HIV-1) [26]. HERV expression has been associated with several autoimmune disorders such as multiple sclerosis [27] and rheumatoid arthritis [28]. Also, HERV transcripts have been found in biopsies from lymphomas, breast cancer, melanoma and teratocarcinomas [29]. Some evidence points to an association of HERV with schizophrenia [30]. With regard to infectious diseases, HERV expression has been shown to be increased in HHV-6 A and B, Epstein-Barr virus (EBV), herpes simplex virus and HIV-1 [31], [32]. The exact role that these associations have on disease mechanisms, if any, remains unclear and in some cases controversial [33].

The HERV-K family of endogenous retroviruses are probably the most recently incorporated, with some inserts having occurred after the rise of hominids [34]. The first association between HERV-K products and HIV-1 infection was reported in 1996 by Löwer and colleagues [35], who reported an observation where there was increase in HERV-K-specific antibodies in HIV-1 infected individuals. Ten years later Contreras-Galindo et al. [31] found that HERV-K RNA plasma expression was higher in HIV-1 infected patients than healthy controls, and that this expression could be induced *in vitro* by HIV-1 infection [36]. Recently, they reported specific HERV-Ks being differentially expressed in HIV-1 infected patients, but not in breast cancer patients, and the presence of molecules that bind HERV-K env antibodies [37]. Increased HERV-K expression was confirmed by our group in a study in which we also identified T cell responses to HERV peptides in subjects with early HIV-1 infection [38]. We have also recently demonstrated that HERV-specific T cells responses persist in chronic HIV-1 infection and are associated with improved virologic control [39]. Interestingly, in addition to HERV-specific responses being highly increased in HIV-1-infected patients compared to healthy donors, within the HIV-1 infected cohort, the responses were much higher in individuals who naturally control HIV replication (“controllers”) than in those who failed to control virus replication and were almost completely absent in patients with advanced disease (as defined by high viral load and low CD4^+^ T cell count). This suggests that anti-HERV-K T cell immunity could play a role in HIV-1 control (reviewed in [40]).

Since immune responses against chronic latent viral infections such as EBV and cytomegalovirus (CMV) can be reactivated during HIV-1 infection and induce immune activation of their cognate T cells [20], and virus-induced activation can be seen in other viral infections such as CMV [41] we hypothesized that HERVs could act in a similar manner and thereby cause an increase in generalized immune activation in HIV-1 infected subjects We focused on HERV-K *env* and *gag* RNA transcripts, since these proviruses have been found to be transcribed in multiple reports, and given their relative new incorporation, are likely to have intact open ready frames. In addition, HERV RNA could interact directly with innate viral recognition through such mechanisms as TLRs in dendritic cells [42]. Therefore, we hypothesized that HERV expression would be a driver of chronic immune activation in individuals with untreated HIV-1 infection.

We found that this hypothesis was refuted by our data, since the relationship was in the opposite direction, and that HERV-K RNA expression was inversely correlated with immune activation in HIV-1 disease.

## Results

### Immune activation levels in HIV-1 infected patients

**Figure 1:**
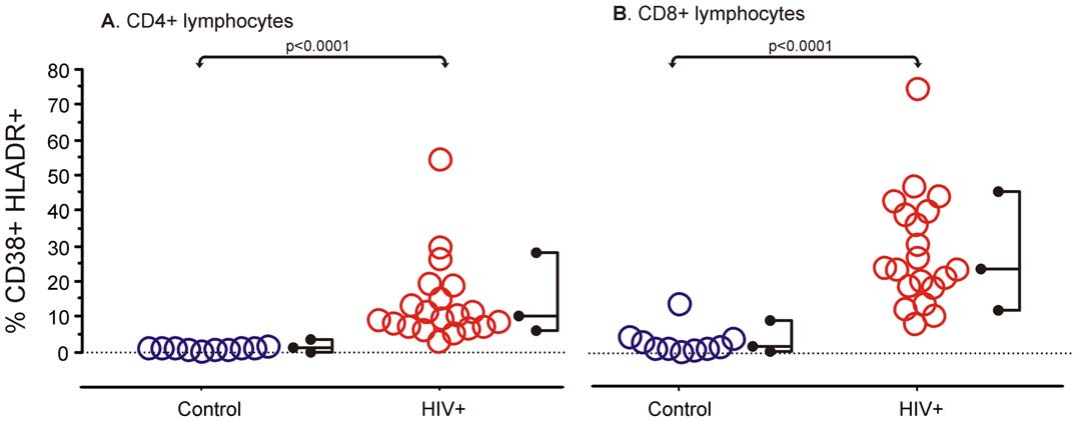
Immune activation as measured by CD38^+^HLADR^+^ T cells, CD4^+^ (A) or CD8^+^ (B). Data for each subject is shown with the right hand fork encompassing 95% of observations and the middle prong at the median. There was significantly increased immune activation in HIV-1-infected patients (n = 20) when compared to controls (n = 10), measured by Mann-Whitney's U.

In a cohort of untreated HIV-1-infected patients at an advanced stage [43], we assessed the level of T cell activation by measuring the percentage of cells coexpressing CD38^+^ and HLA-DR^+^ within the total T cell, and CD4^+^ and CD8^+^ subsets. As expected, T cell Immune activation was significantly higher in HIV-1-infected patients compared to HIV-1-uninfected subjects, as shown by an increased percentage of CD38 and HLA-DR coexpression on both CD4^+^ T cells (p<0.0001) and CD8^+^ T cells (p<0.0001; Figure 1).

### Relative HERV-K RNA expression

**Figure 2:**
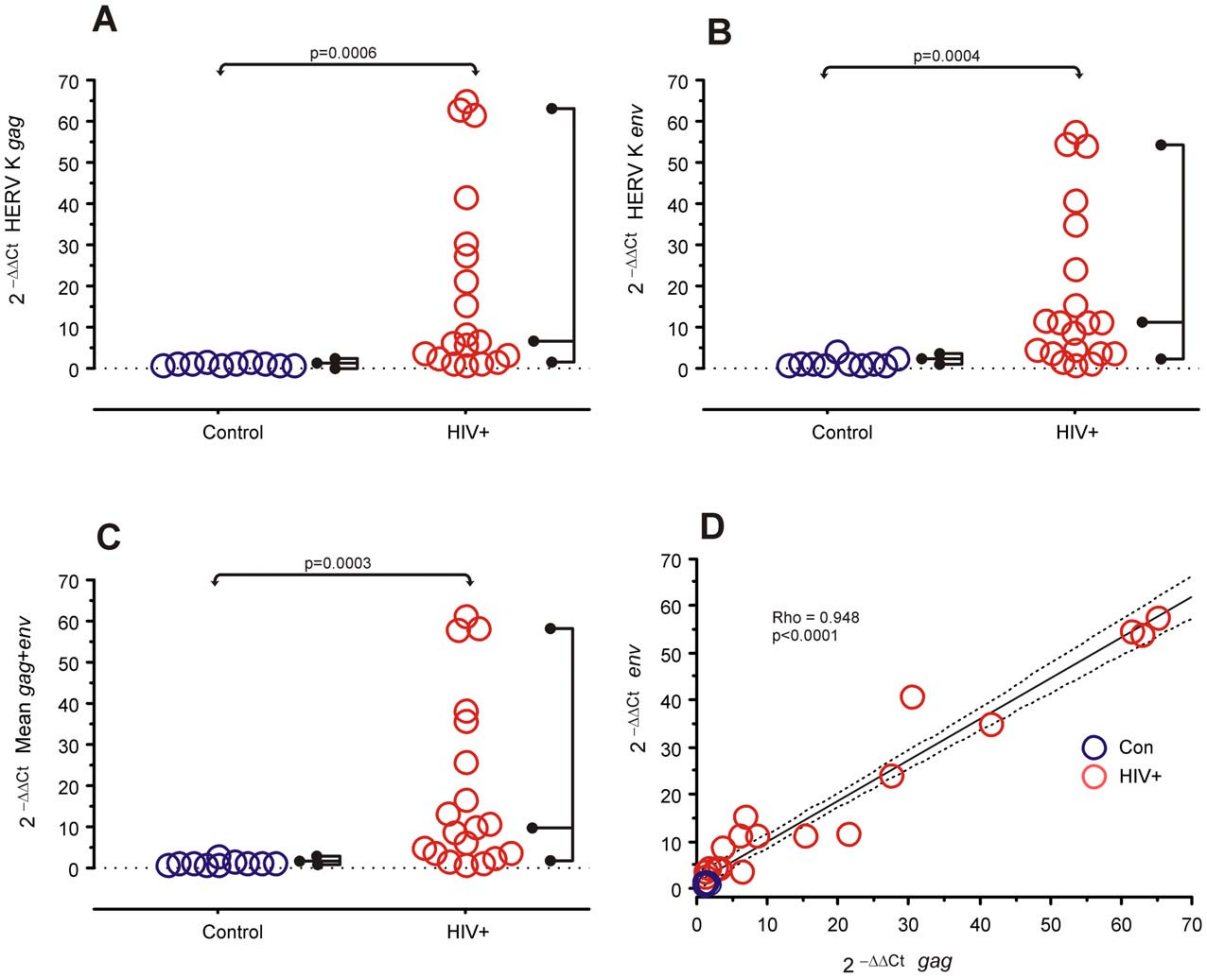
Relative HERV RNA expression in PBMCs in controls (n = 10) and HIV-1 infected subjects (n = 20). A, B and C are data for each subject is shown with the right hand fork encompassing 95% of observations and the middle prong at the median. Expression was calculated as 2^−ΔΔCt^ using the mean of β-actin and G6PDH as housekeeping controls. A. HERV-K *gag*, B. HERV-K *env*, C. The mean expression of *gag* and *env*, which was used in the statistical analysis (Mann-Whitney's U). D. Correlation between HERV-K *gag* and *env* expression. The solid line is the linear regression of the points and dotted lines are the 95% confidence band for the mean. Rho and p values are Spearman rank correlation test.

HERV-K RNA expression levels in peripheral blood mononuclear cells (PBMC) from the HIV-1-infected subjects and healthy controls were assessed by RT-PCR using β-actin and G6PDH as housekeeping reference genes, and measured as 2^−ΔΔCt^ relative expression using each gene as a reference to each other [44]. There was a strong correlation between the expression of both housekeeping genes (Rho = 0.948, p<0.0001), so we used the mean for all calculations. HERV-K *env* and *gag* sequences were compared independently, as well as the mean expression of both. HIV-1-infected patients and controls both had detectable expression of HERV genes, but the HIV-1-infected subjects had a significant increase in the expression of each gene (Figure 2A and B).

There was a strong correlation between the level of expression of each HERV-K gene (Figure 2D), which suggests that both genes are being simultaneously expressed from each proviral HERV-K sequence detected, or at least controlled by the same transcription factor. We used the mean of both HERV genes for further analyses (Figure 1C).

The expression of housekeeping genes was not significantly different between HIV and control patients (

### HERV-K RNA expression and immune activation

**Figure 3:**
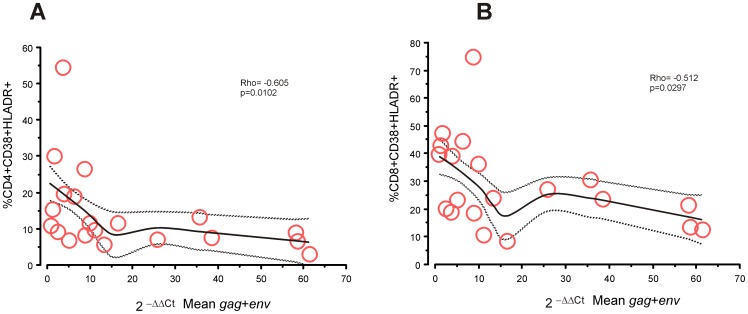
Association between immune activation and HERV RNA expression. Correlation statistics are for HIV-1+ve patients only, Solid lines are the loess smoothing with ± SEM as dotted lines. There was a significantly negative correlation (Spearman's rank correlation) between immune activation and HERV RNA expression in CD4^+^ (A) and CD8^+^ T cells (B).

We next assessed the relationship between T cell activation (as measured by CD38/HLA-DR expression) and HERV-K RNA expression and found a striking negative association between activation of CD4^+^ T cells and HERV-K expression (Figure 3A). There was also a statistically significant inverse correlation between activation of CD8^+^ T cells and HERV expression (Figure 3A and B).

HERV-K expression appeared to be mostly related to the immune activation aspect of HIV-1 disease markers, since it was poorly associated with CD4^+^ T cell total blood count (r = 0.18, p = 0.424) and to plasma HIV viral load (r = −0.15, p = 0.5).

## Discussion

Current models strongly suggest that chronic immune activation in T cells is a strong driver of HIV-1 disease progression. However, the mechanisms of this effect remain largely unexplained. Although HERVs are encoded in the DNA in all cells, their expression appears to be actively modulated in mature cells by methylation and small RNAs (reviewed in [45]), and retrotransposition is inhibited by innate antiretroviral mechanisms such as APOBEC3 [46]. In certain pathological states such as HIV-1 infection, they can be expressed but not recognized as self-antigens, and therefore may elicit an immune response [38] that is analogous to the antiviral response against an exogenous infection. Given that exogenous viruses can play a partial role in exacerbating immune activation [20], we had hypothesized that HERVs could also be an important driver of abnormal activation in chronic HIV-1 disease.

HERV-K family of HERVs are the most recent endogenous retroviruses to be incorporated into the genome [47] and are therefore the most conserved, with several reading open frames in many of the dozens of inserts throughout the genome. One group (HERV-K(HML2)) has 91 insertions of full or near full length proviruses [34]. This makes it a good candidate for expressing mRNA capable of being translated into immunogenic molecules, in addition to being able to be recognized as viral nucleic acid by the innate immune system. Env and Gag peptides are the most T cell immunogenic genes of HIV-1 [48], [49], so we tested the expression of these genes in the HERV-K family.

To address this question, we studied untreated HIV-1-infected individuals who had progressed to advanced or severe immunologic stages of infection [43], with high viral loads and low CD4^+^ T cell counts. As expected, these subjects had increased CD4^+^ and CD8^+^ T cell activation compared to healthy controls. While HERV-K *gag* and *env* expression was present to a low degree in some HIV-1-negative subjects, the infected group had a significantly higher level of relative HERV-K expression, as expected and previously reported [31], [38]. Surprisingly, the HIV-1-infected subjects with the greatest immune activation were among those with the lowest HERV-K expression, suggesting that HERV expression does not contribute to pathologic immune activation in chronic HIV-1 infection.

HERV epitopes have been proposed as potential, non-mutating markers of HIV-1 infected cells that can be targeted and lysed by cytotoxic T cells. The fact that HIV-1-infected individuals with a stronger and broader T cell response against HERV are able to control HIV-1 viremia better than other patients [39] and our present data showing that subjects with less advanced disease (as characterized by abnormal immune activation) included those with increased HERV expression, although many individuals with low HERV-K expression also evidenced lower abnormal immune activation and would suggest that expression of HERV genes and their protein products is associated with a more controlled form of HIV-1 disease.

As HIV-1 infection advances and escapes immune pressures from the host, the immunological state of the infected individual deteriorates. Many studies have shown that in more advanced HIV-1 infection, immune cells express pro-apoptotic markers [9]–[11], the strength and breadth of immune response to HIV-1 antigens decreases [50], immune effector cells are activated even without T-cell receptor (TCR) stimulation [15], and less APOBEC3G is expressed than in recently infected or exposed uninfected individuals [51]. Our previous reports, and the data presented here, support the notion that HERV expression and the immune responses against them are part of a beneficial response that the host mounts against HIV-1 infection, but that is lost when HIV-1 has finally escaped immune pressures and the patient progresses towards AIDS if not treated with antiretroviral drugs. Of note, we have previously shown that individuals who have an undetectable HIV-1 viral load as a result of ART do not have a strong HERV-specific T cell response (in contrast to untreated HIV-1 controllers), suggesting that this response is a potential cause rather than a consequence of good viral control [39].

Figure 3 shows that many patients with low levels of immune activation also have low levels of HERV-K expression, and the current work does not allow further interpretation of this observation. However, future longitudinal studies may test whether these patients have more rapid disease progression than the ones who still express HERV-K.

Interestingly, we observed that HERV-K expression, at least at the level of transcription, was inversely associated with immune activation but not with standard clinical measures of HIV-1 disease status (CD4+ T cell blood count and plasma viral load) in patients with progressive infection. Future studies will focus on examining these relationships in a longitudinal design including earlier time-points prior to CD4+ T cell decline and later time-points to determine the effect of antiretroviral treatment on HERV-K expression and immunity.

There are several possible mechanisms that can be speculated for the inverse correlation between HERV-K expression and immune activation that we observed in this study. HERV transcription has a distinct pattern of expression in the normal state [52], [53] and certain pathological states are thought to disrupt this pattern and cause transcriptional leakage of HERVs [54]. It is possible that HIV-1 causes a similar effect, leading to targeting by cytotoxic T cells [38] or antibodies [37]. As HIV-1 persists and mutates to escape CTL and antibody immune responses, some mutations may also leave the HERV-K inhibition intact, and therefore serve to evade the host's immune system against HIV-1.

It is possible that HERV-K expression leads to the development of HERV-specific cytotoxic T cells that lyse HIV-1-infected cells and thereby indirectly contain immune activation. However, we did not observe high levels HERV-specific CD8^+^ T cells in this group of untreated progressors (reported in [39]), though the peptide set tested was limited and did not completely correspond to the HERV-K *gag* and *env* sequences used to identify mRNA expression. It is also possible that in some individuals, HERV expression leads to the develoment of HERV-specific regulatory T cells (Tregs). Recent literature demonstrating the dependence of murine Treg expansion on endogenous retroviral antigens encoded in the genome also indicates that there may be an intriguing and important role for HERVs in the development of Tregs and their impact on the adaptive immune response to HIV-1 infection [55]. In another scenario, immune activation in advanced HIV-1 disease itself might lead to less HERV-K expression via as yet undefined pathways, though older studies suggest that the opposite is true: activated cells actually express greater levels of HERV-K. Upcoming studies involving larger cohorts of HIV-1-infected subjects with a broad range of natural disease control, including elite controllers, non-controllers and ART-suppressed individuals will be necessary to clearly delineate the relationship between immune activation, HERV-K expression and immunity. We speculate that HERV-K expression could be an alternative mechanism for eliminating HIV-1-infected cells without inducing immune activation (in contrast to HIV-1-specific T cells) given that HERV-K epitopes cannot escape immune responses by mutation as HIV does, and high expression of HERV-K is actually associated with lower activation.

Further studies also should focus on determining the role each cell type in HERV expression, and a systematic analysis of the individual HERVs that are expressed in HIV-1 infection will also be of great interest. The current study forms the basis for future investigations into the complex and important interactions between exogenous and endogenous retroviruses, and the role that HERV might play in HIV-1 pathogenesis.

## Materials and Methods

### Subjects

Demographics of the patients and controls are shown in Table 1. Peripheral blood mononuclear cells (PBMC) from 20 HIV-1-infected patients not on antiretroviral treatment from the San Francisco-based SCOPE cohort were included in this study. We selected patients that were in an advanced or severe immunologic stage, as these would have the highest immune activation. These individuals had a median duration of HIV-1 diagnosis of eight years (IQR  = 5–16 years). Additionally, PBMCs of 10 healthy, HIV-1-negative controls from the Stanford blood donor bank were analyzed. All patients signed informed consent, and the protocol was performed under the SCOPE [56] study, approved by the UCSF Committee on Human Research, and according to the Declaration of Helsinki. Samples were cryopreserved in liquid N_2_ until use.

**Table 1:** Demographic characteristics of the 20 HIV-infected subjects and 10 HIV-uninfected subjects.

*Group*	*CD4+ T cells/μl (Median ± IQR)*	*Viral load (Median copies/ml±IQR)*	*Age (Median yrs ±IQR)*	*Gender*	*Ethnicity*
**HIV+**	200 (144.5–231.5)	151,000 (90,000–231,000)	44 (40.5–48)	15% female	European (50%), African (15%), Latino (15%), Other/mixed (20%)
**Control**	n.d.	n.d.	47 (44–52)	10% female^1^	European (50%), Latino (11%)^2^

1 Gender was not noted in one subject. ^2^Ethnicity was not noted in 4 subjects. n.d. Not determined.

### HERV real-time RT-PCR assay

Two sets of TaqMan primers and probes were designed and used for assessing RNA expression (Table 2). The sequences covered HERV-K env and gag regions. One million peripheral blood mononuclear cells (PBMC) were separated and stored at −80°C in Qiagen RLE buffer with β-mercatoheptanol until processed. RNA was extracted with Qiagen QIAamp RNA Blood Mini Kit following the manufacturer's instructions and at a final 50 μl elution. For preparing the master mix SuperScript® III RT/Platinum® Taq Mix (Invitrogen, Eugene, OR) was used including the optional RNAse inhibitor step. Specific forward and reverse primers for HERV-K gag and env, with their corresponding TaqMan probes. β-actin and G6PDH were used as a housekeeping control to calculate 2^−ΔΔCt^ relative expression. This method allows measuring the relative expression of each gene to an endogenous control, and normalizes measurements [44].

**Table 2:** Primers and probes used in the experiments.

Gene	Forward	Reverse	Probe
HERV-K gag	TCgggAAACgAgCAAAgg	gAATTgggAATgCCCCAgTT	CTCAggCCCCACAAC
HERV-K env	gggTACCTggCCCCATAgAT	CATCATCCCTTCTTCCTCAggTT	ATCgCTGCCCTgCC
β-actin	gAgCGCggCTACAgCTT	TCCTTAATgTCACgCACgATTT	ACCACCACggCCgAgCgg
G6PDH	ATCgACCACTACCTgggCAA	TTCTgCATCACgTCCCggA	AAgATCCTgTTggCAAATCTCAgCACCA

HERV-K sequences were designed on the HERV-K(HML2) group, but are able to bind to several different HERV-K family proviruses.

Two μl of cell associated total RNA from cryopreserved PBMC was amplified in a total reaction volume of 20 μl using the SuperScript™ III Platinum® One-Step Quantitative RT-PCR System (Invitrogen, Carlsbad, CA, USA) with a final concentration of 900 nM of forward and reverse primers, 500 nM of fluorogenic probe and 25 units RNase inhibitor (RNasin®, Promega, San Luis Obispo, CA, USA). Reverse transcription for 30 min at 50°C was followed by a denaturation step of 95°C for 2 min and 45 cycles of 95°C for 15 s and 60°C for 30 s. Reactions were carried out with a StepOne (Applied Biosystems, Foster City, CA) RT-PCR system.

Precision and amplification efficiency of the real-time PCR and RT-PCR assays was also calculated. Within run precision had a standard deviation <0.3 Ct values in all genes tested, and a <0.02 coefficient of variation. The amplification efficiencies of HERV-K gag, HERV-K env and G6PDH and β-Actin assays were determined in triplicate using serial 2-fold dilutions of purified RNA from PBMC from a healthy donor. The efficiencies of these dilutions were compared to each other by assessing the slopes (s) of the curves (threshold cycle versus log dilution). The efficiencies of the assays for *env* and *gag* showed a difference of the slopes (Δs) against the b-actin and G6PDH of 0.057 and 0.121 Ct values, respectively. Validation of the 2^−ΔΔCt^ usage was within the recommended range of <0.1 slope for the ΔCt (0.029 for *gag*, 0.093 for *env*) (data not shown).

### Flow cytometry

PBMCs from all subjects were washed with FACS buffer (phosphate buffered saline with 1% bovine serum albumin, Sigma Aldrich), followed by surface-staining with fluorophore-conjugated antibodies to CD3, CD4, CD8, CD38, and HLA-DR (all BD Biosciences). An amine aqua dye (Invitrogen) was also included to discriminate between live and dead cells. Following staining, PBMCs were washed with FACS buffer, fixed in 1% paraformaldehyde (Polyscience, Niles, IL) and stored at 4°C until analysis. Samples were acquired with an LSR-II system (Becton Dickinson). At least 100,000 events were collected and analyzed with FlowJo software version 9.0 (Tree Star, Ashland, OR). The gating was based on fluorescence minus one (FMO) controls for each fluorophore, applied to all samples equally.

The total number of cells used for the analysis after gating for live cells and CD3+ had a median of 4,731 (IQR 2,422–8752) for CD4+ T cells, and a median of 21,116 (IQR 8,162–32,594) for CD8+. The number of activated cells (CD38+HLA-DR+) captured had a median of 473 (IQR 309–788) for CD4+ T cells and 3,065 (IQR 2,097–7,405) for CD8+ T cells.

### Statistical analysis

All numerical values between groups were tested with Mann-Whitney's test; correlations were tested with Spearman's Rank test. All tests were double tailed and not corrected for multiple comparisons. All analyses were carried out with R statistical software, version 2.10.1.
